# Survival Rate in Hepatocellular Carcinoma after Surgery and Its Association with Clinicopathological Factors: A Single-Center Report

**DOI:** 10.34172/mejdd.2025.441

**Published:** 2025-10-31

**Authors:** Masoomeh Safaei, Farid Azmoudeh-Ardalan, Ali Jafarian, Maede Hoseini, Amir Mohammad Azizpour

**Affiliations:** ^1^Department of Pathology, Cancer Institute, Imam Khomeini Hospital Complex, Tehran University of Medical Sciences, Tehran, Iran; ^2^School of Medicine, Tehran University of Medical Sciences, Tehran, Iran

**Keywords:** Hepatocellular carcinoma, Overall survival, Clinicopathological factors

## Abstract

**Background:**

Hepatocellular carcinoma (HCC) is the sixth most common cancer globally and the third leading cause of cancer-related deaths. Survival rate is a key health indicator that aids in assessing diagnostic and therapeutic approaches. This study aimed to evaluate the survival rate of patients with HCC and identify clinicopathological factors influencing survival in Iran, where data are limited.

**Methods:**

This cross-sectional retrospective study examined the medical records of 45 patients with HCC who underwent surgery at Imam Khomeini hospital, Tehran, from 2013 to 2019. Demographic, clinical, and histopathological data were collected, and survival rates were analyzed using the Kaplan-Meier method. Multivariate analysis was performed using Cox regression to identify independent prognostic factors.

**Results:**

The mean age of patients was 48.6±18.56 years, with 62.2% being male. The average follow-up period was 34.28 months, with a maximum of 102 months. The median overall survival was 26 months, with 1-year and 3-year survival rates of 66.7% and 46.7%, respectively. Multivariate analysis identified older age, tumor size>5 cm, presence of steatosis, history of recurrence, and non-transplantation treatment as independent risk factors for mortality. Other factors, such as tumor stage, histological subtype, and serum alpha-fetoprotein levels, were not significantly related to survival.

**Conclusion:**

Based on the results, mortality in patients with HCC is significantly influenced by factors such as advanced age, larger tumor size (>5 cm), presence of steatosis, prior recurrence, and absence of liver transplantation as a treatment. These findings underscore the limited prognostic value of variables such as tumor stage, histological subtype, and serum AFP levels. Emphasizing the importance of proactive management, regular follow-up of high-risk individuals, and timely detection to facilitate curative interventions appears to be an essential strategy for enhancing survival outcomes in patients with HCC.

## Introduction

 Hepatocellular carcinoma (HCC) is the most common primary liver malignancy and one of the leading causes of cancer-related mortality worldwide.^1^ In terms of global prevalence, liver cancer ranks as the sixth most common malignancy, with a markedly higher incidence in men (ranked fifth) compared with women (ranked seventh).^2^ The incidence of HCC also varies significantly across different demographic and geographical groups, with higher rates seen in men and unmarried individuals relative to women and married patients.^3^ This disease is particularly prevalent in the Middle East,^1^ with the highest incidence observed among individuals aged 70 years and older, and a notably lower occurrence in those under 40.^4^ Globally, approximately 85% of new HCC cases occur in developing countries, with the majority of these cases being diagnosed in Asia.^5^ Various clinicopathological factors, including tumor size, number of tumors, tumor stage and grade, lymph node involvement, surgical margin status, underlying viral infections, and serum alpha-fetoprotein levels, have been associated with prognosis and survival outcomes in patients with HCC.^6-8^

 Surgical resection remains one of the most effective curative treatment options for patients with early-stage HCC who have sufficient liver function reserve. However, recurrence after surgery remains a significant concern, and long-term survival is influenced by multiple pathological and clinical factors.^9^

 However, findings regarding the relationship between these factors and survival outcomes remain inconsistent across different studies, highlighting the complexity of HCC prognosis and underscoring the need for further research to better understand these associations. Understanding the epidemiology, incidence rates, and survival determinants of HCC is crucial for effective disease management and improving patient outcomes. The identification of prognostic factors in different regions is essential for the development of region-specific diagnostic and treatment strategies.^10^ While several studies have identified tumor-related factors that influence survival in HCC,^11^ the complexity of staging, prognosis prediction, and treatment selection in HCC is heightened by the potential impact of liver dysfunction on other organ systems.^12^

 In particular, patients who undergo hepatic resection require careful evaluation of tumor burden, liver function, and surgical margins, as these factors can significantly affect recurrence and overall survival.^9^

 Despite the growing burden of HCC in Asia^13^ and the importance of survival outcomes as a health indicator,^10^ there is a lack of comprehensive research on this topic within the region. This retrospective study aims to evaluate the survival rates of patients with HCC and explore the relationship between various clinicopathological factors and patient survival. By identifying these factors and their associations with survival, we aimed to contribute to the development of region-specific diagnostic and treatment strategies for HCC. These insights could also guide clinical decision-making, improve patient outcomes, and inform public health policies aimed at reducing the burden of liver cancer in affected populations. Furthermore, identifying prognostic markers specific to this region will help develop personalized treatment plans that better address the unique needs of Asian patients with HCC.

 Through this study, we hope to contribute to the broader understanding of HCC epidemiology, risk factors, and survival predictors, ultimately improving outcomes for patients in regions with high disease prevalence and providing a foundation for future research in liver cancer care.

## Materials and Methods

 The design of our study was cross-sectional and retrospective. The aim was to assess the survival rates in patients with HCC and their association with clinicopathological factors among those referred to Imam Khomeini Hospital in Tehran.

 In this study, we reviewed the dataset from the Pathology Department of Imam Khomeini Hospital. The participants were patients diagnosed with HCC who underwent surgery between 2013 and 2019. Paraffin blocks and stained slides using the H&E method from patient samples were retrieved from the pathology department’s archive. Two pathologists re-examined all slides. The histopathological information of the specimens included tumor type, tumor grade, tumor stage, surgical margin status (focal or non-focal involvement), number of involved lymph nodes, vascular and perineural invasion, presence of background liver disease in non-tumoral liver tissue, and presence of background fibrosis in non-tumoral liver tissue.

 Patients were followed up via telephone regarding overall survival, one-year survival, and three-year survival rates. Additionally, clinical information was extracted from the medical records at Imam Khomeini Hospital, including demographic characteristics (age, sex), underlying diseases, tumor size, and number, preoperative serum alpha-fetoprotein levels, type of treatment administered for cancer control, disease recurrence, frequency of recurrences, and hospitalization as potential factors influencing patient survival.

 The data collected from patients in this study included three main sections: clinical data, histopathological data, and survival data. The clinical data consisted of age, sex, underlying diseases, tumor size and number, preoperative serum alpha-fetoprotein (AFP) levels, type of treatment received, disease recurrence status, and the number of recurrences and hospitalizations, all extracted from the patients’ hospitalization records in the Imam Khomeini hospital archive. The histopathological data for the samples under investigation included tumor type, tumor grade, tumor stage, surgical margin status (focal or non-focal involvement), number of affected lymph nodes, vascular and perineural invasion, presence of underlying disease in the non-tumor liver tissue, and the presence of fibrosis in the non-tumor liver tissue. These data were obtained through microscopic examination of hematoxylin and eosin (H&E) stained slides using a light microscope. Information regarding overall survival and 1-year and 3-year survival rates was gathered through follow-up with patients via phone contact.

###  Patient Selection

 The study population consisted of all patients diagnosed with HCC who underwent surgery at Imam Khomeini hospital (Tehran) between 2013 and 2019. Eligible patients were included in the study through a census method, resulting in the inclusion of 45 patients. Patients with incomplete medical records or those for whom follow-up via phone contact was impossible were excluded from the study.

###  Statistical Analysis

 The data collected from patients were coded, and statistical analysis was performed using SPSS software (version 22,

 IBM, Chicago, USA). Quantitative data were reported as mean and standard deviation, while qualitative data were presented as frequency percentages. A significance level of less than 0.05 was considered statistically significant. The normality of the quantitative data was assessed using the Kolmogorov-Smirnov test. For the analysis of variables with normal distribution, parametric tests were used, while non-parametric tests were applied for variables with non-normal distribution. Kaplan-Meier survival analysis was conducted to assess patient survival rates, and multivariate analysis using Cox regression was performed to evaluate the impact of underlying factors on survival outcomes.

 In this study, all provisions of the Helsinki Declaration were considered. Informed consent was obtained from all participants at the time of file creation by the medical records department, allowing the potential use of their medical data for research purposes. The information contained in the patients’ files was kept confidential and was accessible only to the researcher. To ensure patient identity remained anonymous, the data for each case was stored using specific codes.

 The findings include patient survival rates and their correlation with various variables, presented in tables and charts.

## Results

###  Patient Characteristics

 A total of 45 patients diagnosed with HCC were included in this study, comprising 28 men (62.2%) and 17 women (37.8%). The mean age of male patients was significantly higher than that of females (*P* = 0.002) (Supplementary file 1, Table S1). Four patients (8.9%) had non-hepatic comorbidities, including diabetes mellitus, polycythemia vera, tyrosinemia, and renal cell carcinoma. Underlying liver disease was found in 24 patients (53.3%), with hepatitis B virus (HBV) infection being the most prevalent (31.1%). The distribution of underlying liver diseases is shown in Table 1. Histopathologic examination revealed fibrosis or cirrhosis in 24 patients (53.3%) and steatosis in 14 (31.1%).

**Table 1:** Clinicopathological characteristics of patients (n = 45)

**Variable**	**Category/Value**	**Frequency (%)/Value**
Tumor size (cm)	Range	0.6–20
	Median	4.5
	Mean ± SD	6.74 ± 5.3
AFP (ng/mL)	Range	1.3–2561
	Median	7.7
	Mean ± SD	150.56 ± 138.2
Tumor type	Classic	40 (88.9%)
	Fibrolamellar	5 (11.1%)
Number of tumors	1	26 (57.8%)
	2	5 (11.1%)
	3	6 (13.3%)
	> 3	8 (17.8%)
Clinical stage (pT)	pT1	11 (24.4%)
	pT2	20 (44.4%)
	pT3	3 (6.7%)
	pT4	11 (24.4%)
Degree of differentiation	Good	10 (22.2%)
	Moderate	33 (73.3%)
	Poor	2 (4.4%)
Vascular invasion	Yes	31 (68.9%)
	No	14 (31.1%)
Neural invasion	Yes	3 (6.7%)
	No	42 (93.3%)
Lymph node involvement	Yes	2 (4.4%)
	No	43 (95.6%)
Surgical margin involvement	Yes	7 (15.6%)
	No	38 (84.4%)
Type of margin involvement	Focal	6 (85.7%)
	Diffuse	1 (14.3%)
Distance to surgical margin	< 1 mm	5 (13.5%)
	> 1 mm	32 (86.5%)
Underlying liver disease	None	21 (46.67%)
	HBV	14 (31.11%)
	Autoimmune hepatitis	3 (6.67%)
	Budd–Chiari	3 (6.67%)
	HCV	2 (4.44%)
	NASH	2 (4.44%)

SD: Standard deviation; AFP: Alpha-fetoprotein; pT: pathological tumor stage; HBV: Hepatitis B virus; HCV: Hepatitis C virus; NASH: Non-alcoholic steatohepatitis.

 The average tumor size was 6.74 ± 5.3 cm (median: 4.5 cm), and the mean serum AFP level was 150.56 ± 138.2 ng/mL (median: 7.7 ng/mL), as detailed in Table 1. Most tumors were of the classic subtype (88.9%), and 57.8% were unifocal. The majority of patients were diagnosed at stage pT2 or higher, with moderate tumor differentiation in 73.3%. Vascular invasion was observed in 68.9% of cases. Detailed histopathological features and staging are also provided in Table 1.

 Regarding treatment modalities, the most common approach was surgical resection combined with transarterial chemoembolization (TACE) in 15 patients (33.3%), followed by liver transplantation with or without TACE in 18 patients (40%). Treatment frequencies are visualized in Figure S1. During follow-up, recurrence or rehospitalization occurred in 28 patients (62.2%), with 11 patients (24.4%) experiencing it once, 9 (20%) twice, and 8 (17.8%) three or more times.

###  Survival Findings

 The mean follow-up duration was 34.28 ± 27.41 months, and the median survival time was 26 months (range: 0-102 months). One-year and three-year survival rates were 66.7% and 46.7%, respectively. The overall survival rate at the end of follow-up was 44.4%. Survival by sex and presence of underlying liver disease is presented in Table 2. While female patients showed a slightly higher mortality rate than males, the difference was not statistically significant. Among liver disease subtypes, patients with hepatitis C had the highest mortality rate, though this was not significant.

**Table 2:** Survival and mortality analysis by clinicopathological variables

**Variable**	**Category / Value**	**Mortality / Mean±SD**	**Survival / ** **Mean±SD**
Sex	Male	14 (50%)	14 (50%)
	Female	11 (64.7%)	6 (35.3%)
	*P* value	0.336	
Underlying liver disease	Without underlying disease	15 (71.4%)	6 (28.6%)
	Hepatitis B	7 (50%)	7 (50%)
	Hepatitis C	2 (100%)	0
	Autoimmune hepatitis	1 (33.3%)	2 (66.7%)
	NASH	0	2 (100%)
	Budd-Chiari	0	3 (100%)
	*P* value	0.066	
Age	Mean ± SD	52.76 ± 20.34	43.4 ± 14.94
	*P* value	0.033	
Tumor size	Mean ± SD	8.95 ± 5.94	3.99 ± 2.45
	*P* value	0.002	
AFP level	Mean ± SD	138.84 ± 92.5	165.22 ± 80.88
	*P* value	0.163	
Tumor type	Classic	21 (52.5%)	19 (47.5%)
	Fibrolamellar	4 (80%)	1 (20%)
	*P* value	0.350	
Fibrosis	Yes	7 (29.2%)	17 (70.8%)
	No	18 (85.7%)	3 (14.3%)
	*P* value	0.001	
Steatosis	Yes	11 (78.6%)	3 (21.4%)
	No	14 (45.2%)	17 (54.8%)
	*P* value	0.037	
Clinical stage (pT)	pT1	6 (54.5%)	5 (45.5%)
	pT2	8 (40%)	12 (60%)
	pT3	2 (66.7%)	1 (33.3%)
	pT4	9 (81.8%)	2 (18.2%)
	*P* value	0.159	
Degree of differentiation	Good	5 (50%)	5 (50%)
	Moderate	19 (57.6%)	14 (42.4%)
	Poor	1 (50%)	1 (50%)
	*P* value	0.903	
Vascular invasion	Yes	19 (61.3%)	12 (38.7%)
	No	6 (42.9%)	8 (57.1%)
	*P* value	0.249	
Neural invasion	Yes	2 (66.7%)	1 (33.3%)
	No	23 (54.8%)	19 (45.2%)
	P value	0.688	
Lymph node involvement	Yes	23 (53.5%)	20 (46.5%)
	No	2 (100%)	0
	*P* value	0.196	
Surgical margin involvement	Yes	5 (71.4%)	2 (28.6%)
	No	20 (52.6%)	18 (47.4%)
	*P* value	0.358	
Type of margin involvement	Focal	5 (83.3%)	1 (16.7%)
	Diffuse	0	1 (100%)
	*P* value	0.088	
Distance to surgical margin	< 1 mm	4 (80%)	1 (20%)
	> 1 mm	15 (46.9%)	17 (53.1%)
	*P* value	0.168	
Type of treatment	Resection	9 (90%)	1 (10%)
	Liver transplantation	2 (14.3%)	12 (85.7%)
	Resection and TACE	14 (93.3%)	1 (6.7%)
	Transplantation and TACE	0	6 (100%)
	P value	< 0.001	
Recurrence / Rehospitalization	Yes	24 (85.7%)	4 (14.3%)
	No	1 (5.9%)	16 (94.1%)
	*P* value	< 0.001	

SD: Standard deviation; AFP: Alpha-fetoprotein; pT: pathological tumor stage; NASH: Non-alcoholic steatohepatitis; TACE: Transarterial chemoembolization.

 Analysis of continuous variables showed that deceased patients had significantly higher mean age (52.76 ± 20.34 vs. 43.4 ± 14.94 years; *P* = 0.033) and larger tumor size (8.95 ± 5.94 vs. 3.99 ± 2.45 cm; *P* = 0.002) than survivors. AFP levels did not significantly differ between groups (*P* = 0.163) (Table 2).

 Significant associations with mortality were also found for histopathological features. Steatosis was linked to increased mortality (*P* = 0.037), while fibrosis was associated with better survival (*P* = 0.001). No significant differences were observed for tumor type, stage, differentiation, or vascular invasion (Table 2).

 Treatment type and recurrence status were significantly related to patient outcomes (*P* < 0.001). Patients who underwent liver transplantation—with or without TACE—had lower mortality rates than those treated with resection alone or combined with TACE. Recurrence or rehospitalization was strongly associated with reduced survival. These results are illustrated in Figure S2 (Kaplan-Meier survival curve).

 Multivariate Cox regression analysis (Table 3) identified tumor size > 5 cm, presence of steatosis, disease recurrence, and non-transplant treatment as independent predictors of lower survival. Kaplan-Meier survival curves stratified by treatment modality (Figure S3) showed a significant difference in mean survival times based on the type of treatment (log-rank *P* < 0.001). Additional cumulative survival curves based on recurrence status, tumor size, and steatosis are displayed in Figures S4–S6, respectively.

**Table 3:** Results of analysis

**Variables**		**Mean of survival (month)**	**HR**	**95% CI **	***P*** ** value**
Sex	Male	34.03 ± 27.32	1	0.097–1.81	0.243
	Female	34.7 ± 28.4	1.3		
Tumor Type	Classic	37.5 ± 28.16	1	1.20–9.95	0.071
	Fibrolamellar	22.40 ± 12.87	1.58		
Fibrosis	Yes	47.08 ± 30.33	0.34	0.273–5.62	0.180
	No	19.66 ± 13.21	1		
Steatosis	Yes	17.21 ± 14.92	1.73	0.065–0.548	0.002
	No	42 ± 28.43	1		
Clinical stage	pT1	36.81 ± 28.99	1		
	pT2	40.8 ± 31.53	0.73	0.557 - 2.369	0.708
	pT3	25.33 ± 13.79	1.2	0.446–3.133	0.527
	pT4	22.36 ± 16.32	1.5	0.112–4.56	0.169
Degree of differentiation	Good	34.2 ± 23.38	1		
	Moderate	34.87 ± 29.17	1.02	0.548–7.355	0.947
	Poor	25 ± 26.87	1.15	0.755–6.310	0.628
Vascular invasion	Yes	30.8 ± 26.2	1.44	0.645–11.649	0.172
	No	42 ± 29.43	1		
Neural invasion	Yes	17.33 ± 20.79	1.19	0.046–4.346	0.490
	No	35.5 ± 27.62	1		
Surgical margin involvement	Yes	33.85 ± 23.42	1.35	0.347–5.849	0.624
	No	34.36 ± 26.69	1		
Type of margin involvement	Focal	32.5 ± 16.03	3.89	1.123–3.443	0.056
	Diffuse	102	1		
Distance to margin	< 1mm	25.2 ± 20.21	1.7	0.538–3.373	0.316
	> 1mm	36.34 ± 27.76	1		
Tumor number	Single	31.53 ± 27.48	1	0.319–12.025	0.469
	Multiple	38.05 ± 27.61	0.90		
Tumor size	< 5 cm	45.68 ± 30.24	1	1.338–7.408	0.009
	> 5 cm	20.05 ± 14.18	2.22		
Recurrence status	Yes	22.85 ± 24.63	14.4	0.001–0.279	0.004
	No	53.11 ± 20.92	1		
Type of treatment	Transplantation	54.14 ± 26.29	1		
	Resection	18.5 ± 14.1	6.42	1.831–2.296	0.001
	Resection and TACE	15.2 ± 11.11	6.74	1.930–2.043	< 0.001
	Transplantation and TACE	62 ± 22.27	0	0.836–2.323	0.532

HR: Hazard ratio; CI: Confidence interval; pT: pathological tumor stage; TACE: Transarterial chemoembolization.

 Spearman correlation analysis (Table S2) showed a significant inverse relationship between survival duration and patient age and tumor size, while AFP level showed no significant correlation.

## Discussion

 Despite the emergence of various treatment and preventive methods, HCC remains a significant health issue worldwide. Most previous cohort studies on HCC have focused on the epidemiology and risk factors of the disease, with limited research on survival rates and prognostic factors in patients. Therefore, this study investigated the survival rates of 45 patients with HCC who underwent either surgery or liver transplantation, as well as the clinicopathological factors affecting their survival.

 According to the results of this study, the mean survival time of the patients was 34 months, with a median survival of 26 months. The 1-year and 3-year survival rates were 66.7% and 46.7%, respectively. The overall survival rate reported in various studies worldwide shows significant differences. These findings are similar to those of Otto and colleagues, who reported a 1-year survival rate of 62%.^14^ However, the 1-year and 3-year survival rates in a study by Lin in Taiwan were 91% and 72.3%, respectively, which are higher than those in our study.^6^ In general, most studies report lower survival rates. Hassanipour et al in a review study, reported 1-year and 3-year survival rates in Asian countries to be 34.8% and 19%, respectively.^10^ In a study by Lee and colleagues in South Korea, the 1-year and 3-year survival rates were 44.4% and 21%, respectively.^8^ Wang et al in China reported 1-year and 3-year survival rates of 49.3% and 35.3%, respectively.^15^

 In terms of median survival, various studies have shown different results. Some studies in Malaysia have reported very low median survival times of 3.5 and 1.9 months.^16^ On the other hand, studies in Taiwan and Italy have reported higher median survival rates of 26.8 and 25.7 months, respectively,^17,18^ similar to our research. In a separate study in Iran, published in 2019, the mean survival time was 12.1 months,^19^ which was lower than in our study. In this study, patients who underwent tumor resection or chemotherapy were analyzed, while patients undergoing liver transplants were excluded, which may explain the differences.

 Several factors could explain the variations in survival rates between studies, including differences in tumor biology, underlying liver disease conditions, risk factors, and treatment options. Additionally, screening and surveillance for early detection of HCC in high-risk populations could result in higher survival rates in different communities.^20^

 The findings of our study suggest that older age, larger tumor size, presence of steatosis, history of recurrence, and treatment other than transplantation were independent risk factors associated with patient mortality. However, factors such as tumor stage, grade, fibrosis in non-tumoral liver tissue, sex, histological subtype, tumor number, surgical margin involvement, vascular and nerve invasion, and AFP levels did not show a significant correlation with mortality in this study. Tumor size has been reported in many studies as an independent prognostic factor for survival in patients with HCC. In a survey by Sarveazad et al in Iran, tumors larger than 3 cm had a 3.24-fold increased risk of death compared with tumors smaller than 1 cm.^19^ Studies by Lee in South Korea and Wang in China also showed tumor size as an independent prognostic factor.^8,21^ In a study by Tian et al in China, tumor size after surgery was the most crucial factor influencing prognosis.^22^

 In our study, a cutoff point of 5 cm was used as an independent prognostic factor for HCC. This finding highlights tumor size as an important factor in risk stratification and treatment decision-making for patients with HCC.

 In our study, the history of recurrence and rehospitalization was associated with the highest mortality risk. Out of 25 patients who died, 24 (96%) had a history of recurrence. In general, the overall survival after hepatic resection is often compromised by a high frequency of recurrence after surgery.^23^ In two separate studies, the 5-year overall survival rate in patients with recurrence after surgery was 31% and 38%, which were significantly lower than the rates in patients without recurrence, which were 73% and 85%, respectively.^24,25^

 In terms of the patient’s age, the mean age in our study was 48.6 years, with a median of 53 years. This is lower than the mean age reported by Sarveazad et al in Iran (62 years)^19^ and by studies in Egypt (56.5 and 58 years).^26,27^ Our study showed a significant inverse relationship between age and overall survival, with older patients having shorter survival times.

 Regarding the treatment modalities, patients who underwent liver transplantation had significantly longer survival compared with those who underwent resection. Among the 20 patients who received liver transplantations, only two patients died, one from a hepatitis C infection and the other from transplant rejection. All six patients who underwent simultaneous liver transplantation and TACE survived for up to 3 years post-surgery. Studies in the U.S. and China also showed better survival outcomes in patients treated with liver transplantation compared with resection.^22,28^ These findings suggest that liver transplantation may provide a better prognosis for patients with HCC, especially those with advanced liver disease or multifocal tumors.

 Our study also identified steatosis in non-tumoral liver tissue as an independent risk factor for shorter overall survival. However, fibrosis in non-tumoral liver tissue did not show a significant independent correlation with survival. The underlying liver condition plays a crucial role in the development and progression of HCC, with cirrhosis being a considerable risk factor, especially in patients with hepatitis C infection.^29^

 In our study, the male-to-female ratio was 1.65:1, and 62.2% of patients were male. Studies in Egypt, Taiwan, and other countries have also shown higher rates of HCC in men.^6,19,27^ This could be attributed to greater exposure to risk factors, such as androgens, and lower exposure to estrogens, which may have a protective effect against HCC.^30^

 Regarding histopathological factors, including vascular invasion, nerve invasion, lymph node involvement, and surgical margin involvement, no significant correlation with survival was found in our study. However, the small number of cases with lymph node involvement, surgical margin positivity, and nerve invasion may have influenced these results. Several other studies have also shown mixed results, with some indicating a significant relationship between these factors and survival, while others did not.

 Lastly, our study found no significant relationship between AFP levels and overall survival. AFP remains an essential biomarker for diagnosing HCC. However, its usefulness in predicting survival is still debated, as AFP levels can also be elevated in other cancers and chronic liver diseases, including cirrhosis.^31^

 In summary, HCC is a complex disease with various prognostic factors affecting survival outcomes. Further studies with larger sample sizes and more comprehensive data are needed to understand these factors better and improve the management and prognosis of patients with HCC.

 This study has several limitations that should be considered when interpreting the findings. The small sample size may have limited the ability to detect statistically significant differences or trends, particularly in subgroup analyses. Additionally, the retrospective nature of the study makes it susceptible to selection bias, missing data, and limitations in controlling for confounding variables. The fact that the study was conducted at a single center may also limit the generalizability of the results to other populations or healthcare settings.

 Despite these limitations, the study has notable strengths. It focuses on a highly relevant clinical issue in HCC, addressing potential prognostic or therapeutic factors using real-world clinical data. The study also benefits from standardized diagnostic criteria and consistent follow-up protocols within the center, which may reduce variability in outcome assessment. These strengths contribute to the internal validity of the findings and provide valuable insights that can inform future multicenter or prospective research in the field of HCC.

## Conclusion

 Based on the identified prognostic factors, including older age, larger tumor size ( > 5 cm), presence of steatosis, history of recurrence, and non-transplant treatments, continuous monitoring and early diagnosis in high-risk patients with HCC appear crucial for improving prognosis and survival. In contrast, variables such as tumor stage and grade, fibrosis, sex, tumor subtype, number of tumors, surgical margin involvement, vascular/neural invasion, and AFP levels showed no significant association with patient mortality or survival.

## Lay Summary

 The study of people with liver cancer showed that survival rates were better for those who were younger and had smaller tumors. The researchers found that certain factors, like having a history of cancer or extra fat in the liver, made it harder for patients to survive. Early diagnosis and close monitoring can help improve the chances of survival for people at higher risk.

## Supplementary Files

Supplementary file 1 contains Tables S1-S2 and Figures S1-S6.
