# Supplementary material for: Survival Rate in Hepatocellular Carcinoma after Surgery and Its Association with Clinicopathological Factors: A Single-Center Report
Source: Middle East J Dig Dis. 2025 Oct 31;17(4):261–8. doi: 10.34172/mejdd.2025.441 (PMC13150700; doi:10.34172/mejdd.2025.441)
Supplement: Supplementary file 1 — contains Tables S1-S2 and Figures S1-S6. [file mejdd-17-261-s001.pdf]

Middle East J Dig Dis. October 2025;17(4):269-S1

10.34172/mejdd.2025.441

http://mejdd.org

figure S1 : Therapeutic Methods

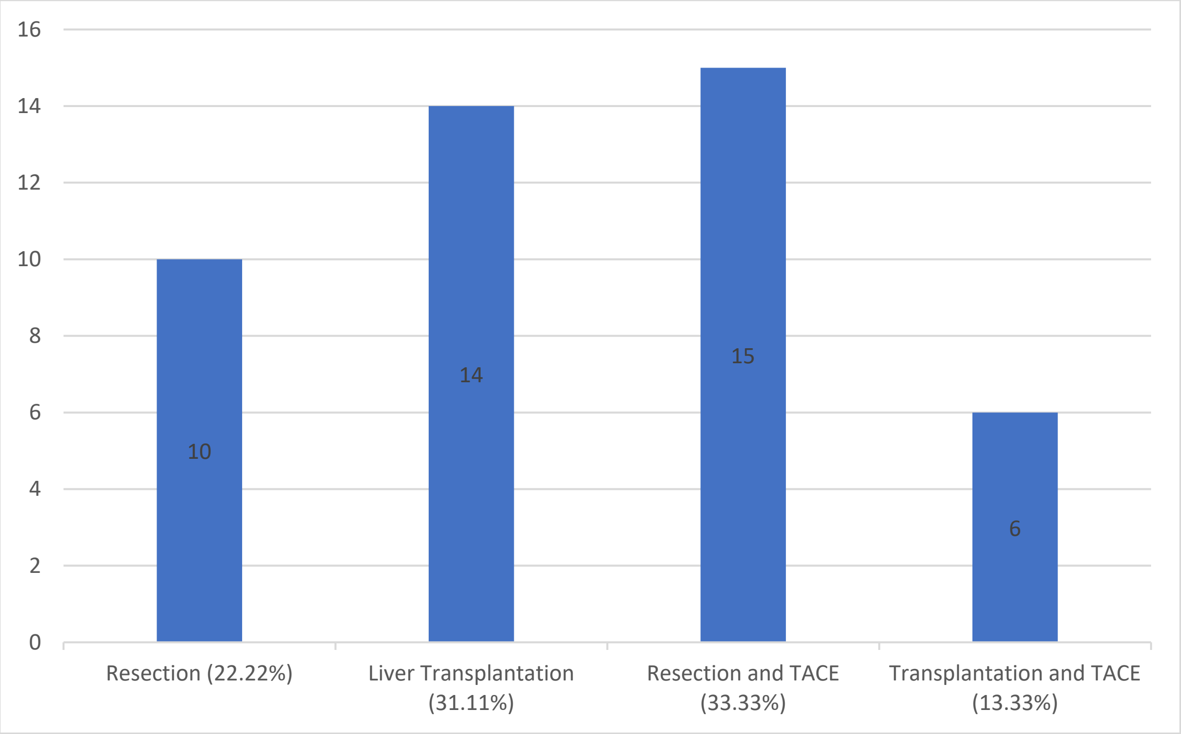

figure S2: The survival rate of patients during the follow up

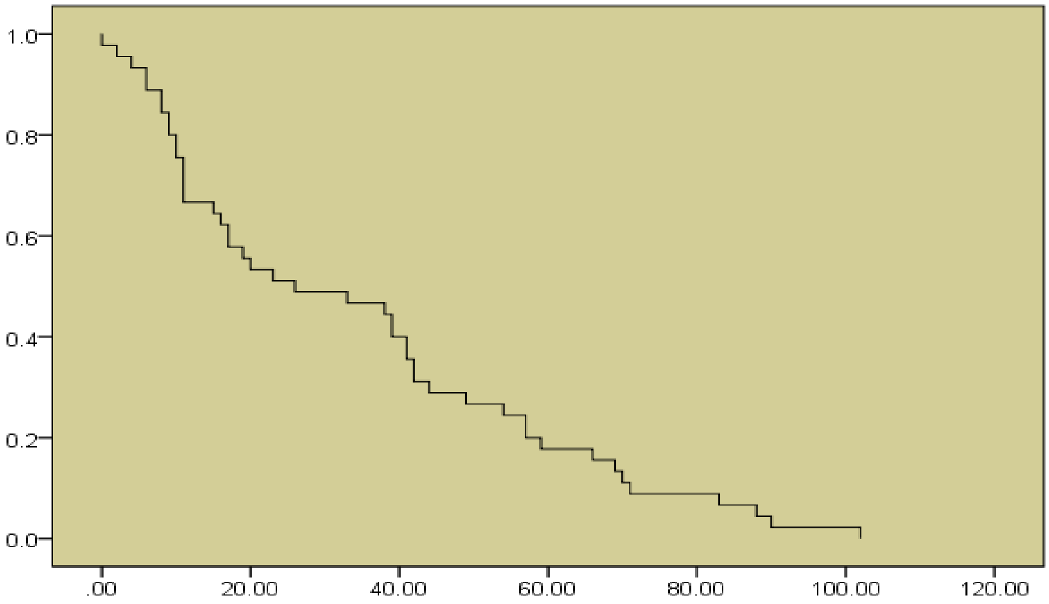

figure S3: Treatment modality

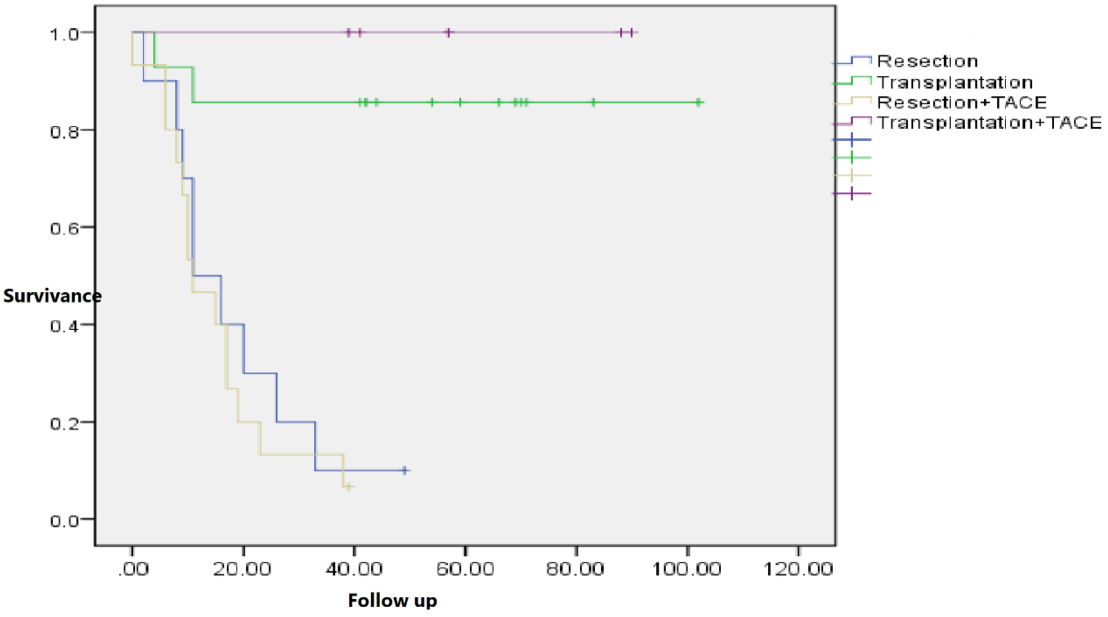

figure S4 : Recurrence

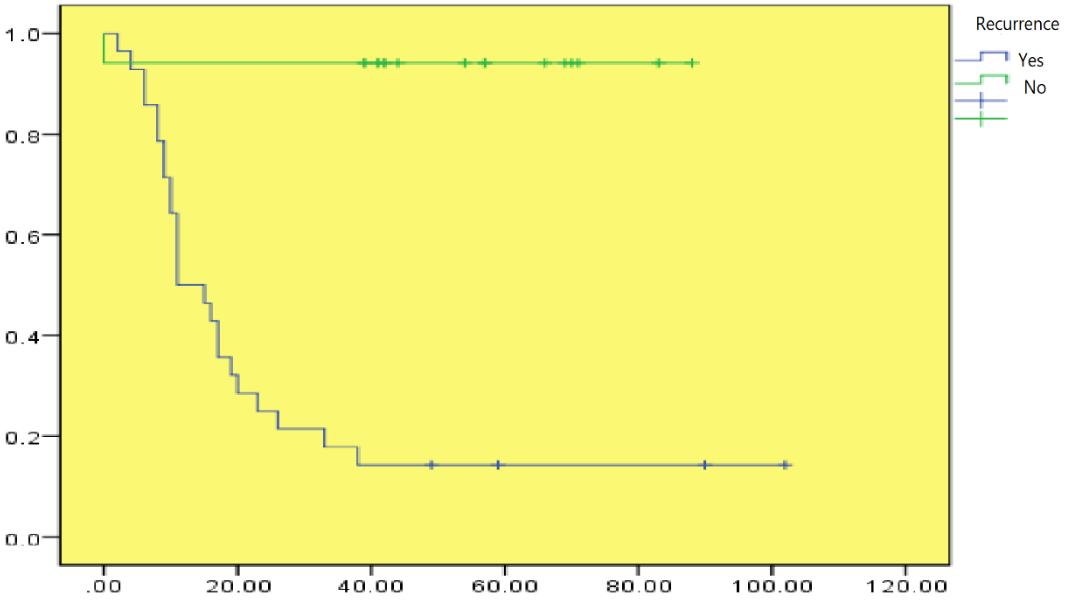

figure S5: Tumor size

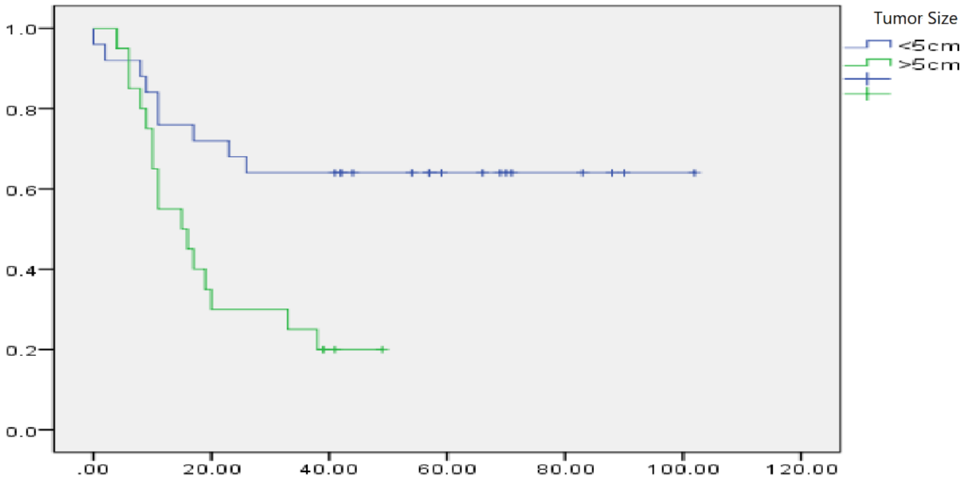

figure S6: Steatosis

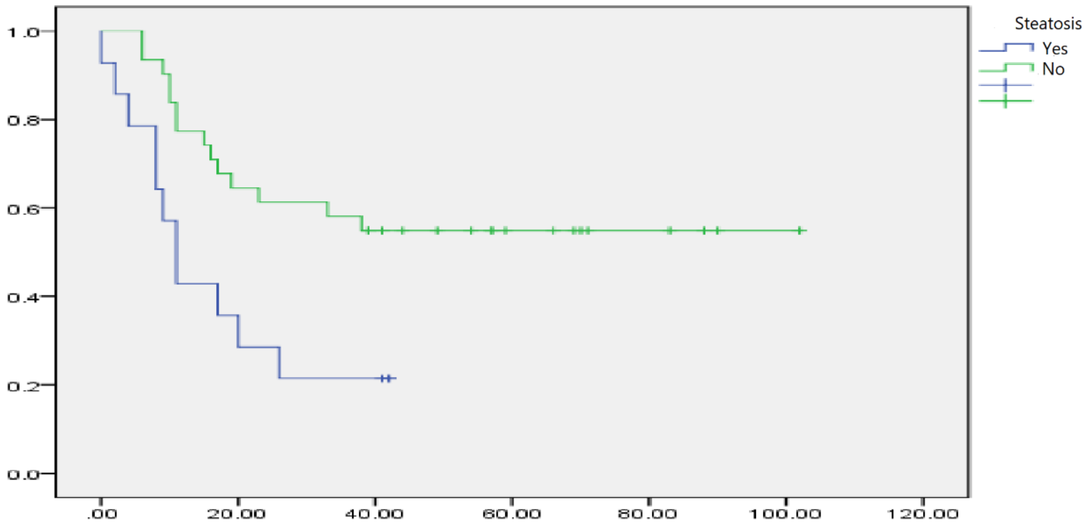

table S1: patient age distribution

Gender / Age	Range (years)	Mean ± SD	Median
male	27-80	55.75 ± 13	53.50
female	11-67	36.82 ± 20.62	29
total	11-80	48.6 ± 18.56	53

table S2: Spearman correlation analysis

Variable	P value	Correlation
Age	0.013	- 0.366
Tumor Size	0.001	- 0.475
AFP level	0.664	- 0.066
